# Quantification of newly produced B and T lymphocytes in untreated chronic lymphocytic leukemia patients

**DOI:** 10.1186/1479-5876-8-111

**Published:** 2010-11-05

**Authors:** Marina Motta, Marco Chiarini, Claudia Ghidini, Cinzia Zanotti, Cinzia Lamorgese, Luigi Caimi, Giuseppe Rossi, Luisa Imberti

**Affiliations:** 1 Department of Hematology, Spedali Civili, Piazzale Spedali Civili 1, 25123, Brescia, Italy; 2 Laboratory of Biotechnology, Diagnostic Department, Spedali Civili, Piazzale Spedali Civili 1, 25123, Brescia, Italy

## Abstract

**Background:**

The immune defects occurring in chronic lymphocytic leukemia are responsible for the frequent occurrence of infections and autoimmune phenomena, and may be involved in the initiation and maintenance of the malignant clone. Here, we evaluated the quantitative defects of newly produced B and T lymphocytes.

**Methods:**

The output of B and T lymphocytes from the production and maturation sites was analyzed in chronic lymphocytic leukemia patients and healthy controls by quantifying kappa-deleting recombination excision circles (KRECs) and T-cell receptor excision circles (TRECs) by a Real-Time PCR assay that simultaneously detects both targets. T-lymphocyte subsets were analyzed by six-color flow cytometric analysis. Data comparison was performed by two-sided Mann-Whitney test.

**Results:**

KRECs level was reduced in untreated chronic lymphocytic leukemia patients studied at the very early stage of the disease, whereas the release of TRECs^+ ^cells was preserved. Furthermore, the observed increase of CD4^+ ^lymphocytes could be ascribed to the accumulation of CD4^+ ^cells with effector memory phenotype.

**Conclusions:**

The decreased number of newly produced B lymphocytes in these patients is likely related to a homeostatic mechanism by which the immune system balances the abnormal B-cell expansion. This feature may precede the profound defect of humoral immunity characterizing the later stages of the disease.

## Background

Profound defects of both humoral and cell-mediated immunity have been described in patients with chronic lymphocytic leukemia (CLL), a disease characterized by the accumulation of mature, malignant, monoclonal B lymphocytes in blood, lymph nodes, spleen, liver, and bone marrow [1]. The disease is characterized by the presence of immune defects, responsible for the frequent occurrence of infections and autoimmune phenomena, that may be involved in the initiation and maintenance of the malignant clone. The immune abnormalities include reduced immunoglobulin (Ig) levels, as well as qualitative and quantitative defects of B, T, NK cells, neutrophils, and the monocyte/macrophage lineage [2,3]. All these immunological changes are linked to an increased frequency and severity of infections [3]. Since CLL represents a heterogeneous disease with a very variable outcome, a reliable prognosis at the time of initial diagnosis is difficult to predict; similarly, only few early markers anticipating the immune defects arising in the later stages of the disease have been up to now identified. In this context, a small size of the blood T/NK-cell compartment compared to that of circulating leukemic clone at the time of diagnosis was associated with more advanced stages, raising the possibility that CLL patients with efficient host immunity may experience a more indolent disease due to a more effective immune response against the disease [2]. However, the maintenance of an immune surveillance needs a continuous source of newly produced B and T lymphocytes. While it has been found that the proliferation of malignant B cells decreases the number of newly mobilized T cells from the thymus [4], it is not known whether this may also influence the release of new B cells from the bone marrow. To answer this question, we combined the method of kappa-deleting recombination excision circles (KRECs) detection, initially developed by van Zelm *et al *[5] and modified later by Fronkova *et al *[6], with the well established method of measuring T-cell receptor excision circles (TRECs) [7], thus obtaining a duplex Real-Time PCR assay allowing the simultaneous measure of newly produced B and T cells. KRECs and TRECs are episomal DNA products generated during the lymphocyte development and differentiation process, when B- and T-cell receptor gene rearrangements occur and specific chromosomal sequences need to be excised [5-7]. These excision products cannot be replicated and, therefore, KRECs and TRECs are diluted when cells proliferate, and are lost when cells die. Since KRECs are randomly present in about 50% of B cells released from the bone marrow and TRECs in 70% of T cells leaving the thymus, their quantification is considered a reliable estimate of the amount of newly produced B and T lymphocytes [8,9]. Here, we applied the new assay, together with the flow cytometry, to quantify the number of recently produced B and T cells and the peripheral lymphocyte expansion in untreated CLL patients, who were at a very early stage of the disease.

## Methods

### Patients

Peripheral blood from 12 untreated CLL patients (male:female ratio: 5:1, median age: 66 years, and range: 48-77 years) who attended the outpatient clinic of our Institution and from 20 age-matched healthy controls (male:female ratio: 5:2, median age: 65 years, and range: 50-69 years) was used for flow cytometric analysis and for peripheral blood mononuclear cells (PBMC) preparation by Ficoll-Hypaque gradient centrifugation. The participants, who were prospectively enrolled from November 2007 to September 2009, signed an informed consent; all experimental procedures, performed on samples collected from 1 to 134 months after the diagnosis, were done according to Helsinki declaration, as requested by our Institutional Ethical Committee (resolution n° 512 of June 25, 2007). DNA was obtained from PBMC and from a human lymphoblastoid B-cell line using the QIAamp DNA Blood Mini Kit (Qiagen).

Blood samples were also sent to the laboratory for routine tests, which included the immunophenotyping of peripheral blood required for the diagnosis of CLL as well as prognostic tests such as serum β2-microglobulin and Ig determination, fluorescence in situ hybridization (FISH) analysis for del13q14, del17p13, and del11q22-q23, +12, and sequence study of rearranged immunoglobulin heavy chain variable (IgVH) gene mutational status.

### Characterization of T-cell subpopulations

The monoclonal antibodies used for six-color flow cytometric analysis were purchased from BD Pharmingen (fluorescein isothiocyanate anti-CD3 and -CD45RA, peridin-clorophyll protein-Cy5.5 anti-CD8 and allophycocyanin-H7 anti-CD4), BioLegend (phycoerythrin anti-CD25 and peridin-clorophyll protein-Cy5.5 anti-CCR7), eBioscience (phycoerythrin-Cy7 anti-CD127), and Miltenyi Biotech (allophycocyanin anti-CD31). ^thymic^naive Th cells were defined as CD4^+ ^T helper (Th) cells with naive (CD4^+^CD45RA^+^CCR7^+^) phenotype also expressing CD31^+ ^molecule, T regulatory cells (Treg) as CD4^+^CD25^int/high^CD127^low/- ^lymphocytes [10,11], and ^thymic^naive Th cells-Treg as Treg expressing CD45RA, CCR7, and CD31 markers [12]. Effector memory (T_EM_) and central memory (T_CM_) T cells were lymphocytes displaying CD4^+^CD45RA^-^CCR7^- ^and CD4^+^CD45RA^-^CCR7^+ ^phenotype, respectively [11]. For the quantification of ^thymic^naive Th cells and Treg within peripheral blood, CD4^+ ^cells were first gated on lymphocytes and then analyzed for the expression of other surface antigens. CD3^+^CD8^+ ^cytotoxic T lymphocyte (CTL) population was evaluated in a separate tube. Data were collected on a FACSCanto II cytometer and results were analyzed with FACSDiva software (BD Biosciences).

### Real-Time PCR for KRECs and TRECs quantification

The number of KRECs and TRECs was simultaneously quantified with a duplex quantitative Real-Time PCR protocol performed on the 7500 Fast Real-Time PCR and data were analyzed by 7500 Fast Real-Time System Software (Applied Biosystems); the amplification of the reference gene, a segment of T-cell receptor constant alpha chain (TRAC), was done in the same plate. The sequences and the quantity of primers and probes used for the assay, as well as the amplification schedule, were described elsewhere [13,14]. KRECs, TRECs, and TRAC copy number has been obtained by extrapolating the respective sample quantities from the standard curve obtained by serial dilutions (10^6^, 10^5^, 10^4^, 10^3^, 10^2^, and 10) of a linearized plasmid DNA, containing three inserts corresponding to fragments of KRECs, TRECs and TRAC.

The number of KRECs or TRECs (copies/PBMC) is calculated with the following formula:

(1) $$\frac{\text{mean of KRECs or TRECs quantity}}{\text{mean of TRAC quantity / }2}$$

The mean quantity of TRAC has to be divided by 2 because each cell carries two copies of TRAC gene, i.e., one for each chromosome.

Results were expressed either as copies/10^6 ^PBMC or copies/mL obtained respectively by multiplying the above calculated value by 10^6^, or, as done by Chen *et al *[15], by the number of lymphocytes plus monocytes (which are the cells obtained in PBMC preparation).

Finally, the average number of B-cell divisions was evaluated, as reported by van Zelm *et al *[5], by calculating the difference between the cycle threshold number obtained by PCR amplification of signal joints, which are sequences contained into KRECs, and the cycle threshold number obtained after amplification of coding joints, which are sequences generated during the rearrangement of IGK chain that remain stably present in the genome and are duplicated during each cell division.

### Statistical analysis

Since data did not follow a Gaussian distribution, they were described in terms of median and interquartile range, and comparisons were performed by two-sided Mann-Whitney test. Results were considered significant if P < 0.05.

## Results and Discussion

### Characterization of CLL patients

All patients enrolled in this study were in a very early stage of disease (Rai stage 0, Binet stage A) and had not been previously treated. Their demographic and laboratory parameters are shown in Table 1. The analysis of biological prognostic factors showed 7 (58%) patients with mutated IgVH, 6 (50%) patients with 13q14 deletion at FISH analysis, and 3 (25%) patients with β2-microglobulin above the normal range. A decrease in serum Ig levels during the course of the disease is a common feature of CLL and correlates with the disease stage and the occurrence of infections [3]. Accordingly, in all our patients but one, the IgG and IgA serum levels were within the normal range found in controls, and this was expected, considering their very early stage of disease. On the contrary, IgM level was below the normal range in 7 (58%) patients, thus indicating that the reduced concentration of IgM is not only the most frequent Ig alteration observed in CLL [16], but likely also the most precocious.

**Table 1:** Demographic, clinical and laboratory parameters of CLL patients

Patients	*1*	*2*	*3*	*4*	*5*	*6*	*7*	*8*	*9*	*10*	*11*	*12*	Controls (range)
Age	68	65	69	68	48	77	73	53	67	53	56	66	50-69
Gender	M*	M	M	M	M	M	M	F	M	M	M	F	na
Rai stage	0	0	0	0	0	0	0	0	0	0	0	0	na
Binet stage	A	A	A	A	A	A	A	A	A	A	A	A	na
Lymphocytes/μL	12 350	30 210	27 500	8 050	5 290	38 470	47 810	14 330	6 030	10 980	24 680	11 360	950-4 612
Haemoglobin (g/dL)	15.6	13.4	14.0	14.2	14.4	13.5	11.7	15.5	16.0	15.2	14.5	14.2	14-18
Platelets (10^3^/μL)	236	216	173	128	247	211	139	160	150	147	220	183	130-400
β2-microglobulin (mg/L)	2.0	2.5	2.8	2.2	1.9	2.1	4.7	2.0	3.7	2.4	2.5	2.4	< 2.5
Direct Antiglobulin Test	neg	neg	neg	neg	neg	neg	neg	neg	neg	neg	neg	neg	neg
IgVH mutational status	mut	unm	mut	unm	mut	unm	mut	unm	mut	unm	mut	mut	na
FISH	del13q14	neg	del13q14	neg	neg	del13q14	del13q14	del13q14	neg	neg	neg	del13q14	na
IgG (mg/dL)	979	1 104	936	857	740	908	672	551	702	904	860	1 448	690-1 500
Clonally expanded chains	Igλ	Igκ	Igκ	Igλ	Igλ	Igλ	Igκ	Igκ	Igκ	Igκ	Igκ	Igκ	na
IgA (mg/dL)	190	368	107	186	113	86	234	40	123	211	243	106	85-410
IgM (mg/dL)	38	58	38	53	113	15	36	36	17	46	13	95	40-240

*Abbreviations: M, male; F, female; na, not applicable; neg, negative; IgVH, immunoglobulin heavy chain variable genes; mut, mutated; unm, unmutated; FISH, fluorescence in situ hybridization.

### Analysis of tumor DNA interference in KRECs and TRECs quantification

**Figure 1:**
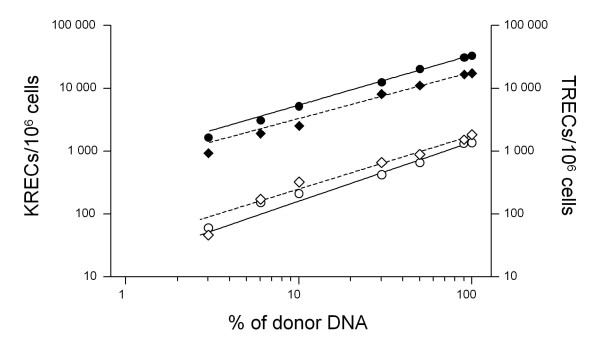
**KRECs and TRECs determination in increasing concentrations of non-tumoral DNA into DNA from a lymphoblastoid B-cell line**. DNA extracted from two healthy controls with either high (filled symbols) or low (open symbols) number of KRECs (circles) and TRECs (diamonds) was diluted into DNA extracted from a lymphoblastoid B-cell line, in order to obtain decreasing concentration of tumoral DNA. Straight line: regression line for KRECs; dotted-line: regression line for TRECs.

To exclude the potential confounding effect of tumor DNA derived from monoclonal B cells on the quantification of KRECs and TRECs, genomic DNA from PBMC of 2 healthy donors with high and low number of KRECs and TRECs was serially diluted into DNA of a human lymphoblastoid cell line to obtain final concentrations of normal lymphocyte DNA ranging from 3% to 100%. While KRECs and TRECs were undetectable in 100% tumor DNA, the amount of KRECs/10^6 ^and TRECs/10^6 ^cells of both donors showed a linear change, being detected even at concentration as low as 3% of normal DNA (Figure 1), suggesting that the presence of high number of blasts in CLL patient samples should not bias the assay results.

### Quantification of newly produced B cells and measure of the average number of B-cell divisions in CLL patients

While the decreased Ig synthesis in CLL has been previously ascribed to the release of inhibitory cytokines upon cell-cell contact between normal and malignant B cells [3], the finding of an early IgM decrease could be also due to changes in the profile of different B-lymphocyte subpopulations, as demonstrated in patients with selective IgM deficiency [17]. Indeed, we found that another B-cell compartment defect observed in CLL patients was the significant decrease of KRECs, both measured per 10^6 ^PBMC and per mL of blood (Table 2). It is noteworthy that to perform KRECs analysis it is not necessary to separate normal from leukemic population since KRECs are not contained in B lymphocytes that have undergone multiple divisions, like clonally-derived leukemic cells. Therefore, if the low number of KRECs/10^6 ^PBMC could be ascribed to the altered proportion of normal B cells that was greatly reduced due to the expansion of leukemic cells, the decreased number of KRECs/mL clearly indicated a real decline in newly produced B lymphocytes in the patients compared to controls. This result suggests that one of the reasons of the early IgM decrease could be attributed to the reduced production of new B lymphocytes because if Ig production is not sustained by a continuous supply of new B cells, Ig synthesis would progressively decrease as the old B cells die off. When we compared the number of KRECs of patients with low and normal IgM serum level, we did not find a significant difference, likely because of the low number of patients included in the two groups. Analogously, there was no significant correlation between the number of lymphocytes and the number of KRECs/mL. This negative result could be ascribed to the wide range not only of lymphocytes of our CLL patients, which was between 5 000 and 48 000 cells/μL, but also to the KRECs number, which varied greatly between individuals [[13] and unpublished observation].

**Table 2:** Number of KRECs and TRECs and average number of B-cell divisions

		**Patients**		**Controls**		
				
		***median***	IQR*	***median***	***IQR***	
						
						
KRECs	/10^6 ^PBMC	200	99-448	5 372	2 798-7 617	**P = 0.0001**
	/mL	3 763	1 318-6 486	12 942	6 556-19 490	**P = 0.0001**
						
Average number		6.7	3.8-14.1	4.0	3.0-4.5	**P = 0.003**
of B-cell divisions						
						
						
TRECs	/10^6 ^PBMC	216	64-949	1 374	834-3 046	**P = 0.002**
	/mL	2 869	1 601-11 812	3 053	1 960-6 401	**NS**

KRECs and TRECs were determined by Real-Time PCR. Results are given both as copies/10^6 ^PBMC and copies/mL. The average number of B-cell divisions was calculated as the difference between the cycle threshold number obtained by PCR amplification of signal joints, and the cycle threshold number obtained after amplification of coding joints.

*Abbreviations: IQR, Interquartile range; KRECs, kappa-deleting recombination excision circles; TRECs, T-cell receptor excision circles.

As expected, the average number of B-cell divisions, determined according to van Zelm *et al *[5], was significantly increased in our CLL patients (Table 2). The presence of coding joints in all Igλ^+ ^mature B lymphocytes and only in about 30% of Igκ^+ ^B cells is the reason of the lower average number of B-cell divisions found in patients with clonal expansions of Igκ chains (see Table 1). However, 3 (25%) of these patients (Pt 1: 4.5, Pt 3: 3.6 and Pt 7: 3.2 average number of B-cell divisions) showed the highest number of KRECs (Pt 1: 6 472/mL, Pt 3: 8 513/mL, and Pt 7: 7 396/mL).

### Quantification of newly produced T cells and phenotypic analysis of T-cell subpopulations

We then investigated if B-cell lymphocytosis may also affect the extent of new T-lymphocyte production. Similarly to what observed by Nardini *et al *[4], we found that the median number of TRECs/10^6 ^PBMC was significantly lower in CLL patients than in controls (Table 2). Analogously to that reported for KRECs, the interpretation of results expressed as TRECs/10^6 ^PBMC can be objectionable because the increased number of peripheral divisions sustained by tumor cells artificially dilutes the TRECs level, regardless of recent thymic production. On the contrary, TRECs number calculated per mL of blood is considered to be more reliable of thymic function, especially when significant cellular proliferation occurs [18]. Indeed, we found that when calculated per mL of blood, the median number of TRECs was comparable in CLL patients and controls. This result is supported by the presence in both groups of a similar number of naive lymphocytes and, within this subset, of comparable number of ^thymic^naive Th cells, which are known to represent the fraction of lymphocytes recently emigrated from the thymus (Table 3) [19]. Likewise, similar values of Treg and ^thymic^naive-Treg were found in patients and controls. Therefore, we have not found in these CLL patients at the very early disease stage the decreased number of Treg observed by Beyer *et al *[20]. This discrepancy may be due to the fact that these authors preferentially analyzed patients at later disease stage (Binet stage B and C), and because they identified Treg as CD4^+^CD25^high ^cells while, according to Liu *et al *[10], we more finely targeted this subpopulation by including in Treg subset only CD4^+^CD25^int/high^CD127^low/- ^lymphocytes. T_CM _cell number was not different in CLL patients and controls, while the percentage and number of T_EM _cells were higher in the patients. The expansion of these cells, which lacking CCR7 expression have the capacity to migrate to inflammation sites and to produce large amounts of proinflammatory cytokines, may be one of the reasons of the increased number of CD4^+ ^Th cells that we have found in our patients (Table 3), which is known to be a common characteristic of CLL patients [3]. The observed skewing towards T_EM _is likely related to a strong and persistent tumor antigenic trigger, and is not linked to homeostatic proliferation due to previous exposure to immunosuppressive drugs, since our patients were all untreated. Finally, while the percentage of CTL was significantly lower in these patients, the total number of this cell population was comparable to that of controls.

**Table 3:** Phenotypic characterization of T-cell subpopulations

		**Patients**		**Controls**		
				
		***median***	IQR*	***median***	***IQR***	
						
Th cells	%	10.2	4.7-23.9	50.9	43.5-54.7	**P = 0.002**
	cells/μL	1 585	1 275-2 533	1 029	785-1 428	**P = 0.05**
						
naive Th cells	%	46.4	27.2-49.5	51.1	46.5-62.5	**NS**
	cells/μL	715	381-834	533	363-786	**NS**
						
^thymic^naive Th cells	%	54.4	41.9-63.6	64.1	58.4-70.1	**NS**
	cells/μL	345	228-447	333	223-545	**NS**
						
Treg	%	4.8	3.3-6.1	5.4	4.7-7.4	**NS**
	cells/μL	82	44-123	62	44-80	**NS**
						
^thymic^naive-Treg	%	2.0	1.5-3.0	1.8	1.0-2.9	**NS**
	cells/μL	8	4-17	7	4-11	**NS**
						
T_EM_	%	22.4	10.9-31.9	10.4	8.0-11.5	**P = 0.04**
	cells/μL	245	202-367	98	80-146	**P = 0.0002**
						
T_CM_	%	30.8	24.1-40.1	30.7	26.7-37.8	**NS**
	cells/μL	520	270-957	308	248-401	**NS**
						
CTL	%	4.1	3.0-9.0	24.8	22.2-29.0	**P < 0.0001**
	cells/μL	479	350-780	483	387-539	**NS**

T-cell subpopulations were determined by six-color flow cytometric analysis using various combinations of monoclonal antibodies. The percentage of Th cells and CTL is obtained after gating on lymphocytes, that of naive Th cells, Treg, T_EM _and T_CM _after gating on Th cells, and that of ^thymic^naive Th cells after gating on naive Th cells. The percentage of ^thymic^naive Treg is obtained after gating on ^thymic^naive Th cells. *Abbreviations: IQR, Interquartile range; Th cells, T helper cells; Treg, regulatory T cells; T_EM_, effector memory T cells; T_CM_, central memory T cells; CTL, cytotoxic T cells.

## Conclusions

Based on these preliminary observations we suggest that the production of new T lymphocytes is normal in CLL at the very early disease stage; the presence of CD4 lymphocytosis can be partially ascribed to the accumulation of CD4^+ ^effector memory cells in the peripheral blood. On the contrary, the number of newly produced B cells is precociously reduced and this may represent a warning signal anticipating the profound defects of humoral immunity, which normally characterize the later stages of the disease. Therefore, we are currently following patients at later stages of the disease in order to investigate modifications of newly produced B and T lymphocytes in the course of the therapy.

## Competing interests

The authors declare that they have no competing interests.

## Authors' contributions

LI was the principal investigator and takes primary responsibility for the paper. MM and GR recruited the patients. MC, CG, CZ and CL performed the laboratory work for this study. LI, MM, LC and GR wrote the manuscript and participated to the discussion. All authors read and approved the final manuscript.
